# Quality of information provided by Brazilian Fertility Clinic websites: Compliance with Brazilian Medical Council (CFM) and American Society for Reproductive Medicine (ASRM) Guidelines

**DOI:** 10.5935/1518-0557.20220026

**Published:** 2023

**Authors:** Márcia Mendonça Carneiro, Caio Nobuyoshi Koga, Marcela Chagas Lima Mussi, Pollyanna Faria Fradico, Márcia Cristina França Ferreira

**Affiliations:** 1 Departamento de Ginecologia e Obstetrícia, Faculdade de Medicina da UFMG, Belo Horizonte, MG, Brazil; 2 ORIGEN Centro de Medicina Reprodutiva, Belo Horizonte, MG, Brazil

**Keywords:** *In vitro* fertilization, IVF, ART, Internet, infertility, quality, advertising, guidelines, success rate

## Abstract

**Objective:**

To evaluate the websites of Brazilian fertility clinics included in the 11th Report of the National Embryo Production System (SisEmbrio, 2017) for compliance with the 2004 American Society for Reproductive Medicine (ASRM) and the Brazilian Medical Council (Conselho Federal de Medicina, CFM) guidelines for advertising.

**Methods:**

We performed an online evaluation of the websites of clinics listed in the 11th SisEmbrio report based on criteria from the 2004 ASRM guidelines (publication of success rates, live birth rates (LBR), method of LBR calculation, success rates by age range and diagnosis, experimental/investigational nature of procedures and the practice of comparison marketing) and CFM guidelines (clinic director name and register visible on the website; no prices displayed, no photos of patients nor success stories with patient identification).

**Results:**

A total of 161 SiSEmbrio-registered clinics were evaluated: 153 (95.0%) had functional websites, and only seven were public clinics. Social media presence was as follows: 87 (54.03%) were on WhatsApp; 128 (79.5%) were on Facebook; and 122 (75.8%) were on Instagram. Seventy-five (46.6%) were on other social media platforms (YouTube, LinkedIn, and Twitter). Regarding CFM recommendations, 49 (30.4%) showed information of a registered director, 85 (52.8%) showed patient photos on their websites and/or social media accounts. Fifty-four clinics published success rates (33.5%) and 19 (11.8%) used their own data, whereas seven (4.3%) showed pregnancy rates by age. None reported LBR or advertised prices.

**Conclusions:**

The information published online by Brazilian fertility clinics is heterogeneous in nature. A significant portion of the websites does not follow some of the ASRM and CFM guidelines for advertising.

## INTRODUCTION

Infertility, defined as absence of pregnancy after twelve months of unprotected intercourse, has a reported prevalence of 9 to 18% in the general population and is estimated to affect 48.5 million couples around the world (Mascarenhas *et al*., 2012).

*In vitro* fertilization (IVF) has been successfully used since 1978 and millions of babies have been born worldwide. Success rates depend mostly on maternal age, but other factors such as diagnosis, response to treatment, and treatment method may also influence pregnancy rates (Luke *et al.*, 2012). Over the years, IVF has become the standard treatment for millions of infertile couples all over the world. As IVF indications and success rates grew, so did the assisted reproduction market and the number of clinics, making it a relatively privatized and lucrative sector in medicine.

In Brazil, there are 53 million women of reproductive age (15-49 years of age) and about four million infertile couples (IBGE, 2012). Unfortunately, only a part of these couples has access to advanced treatments such as IVF. In Brazil, such programs must be registered and adhere to National Health Surveillance Agency (ANVISA) guidelines. In 2005, the Agency created the National Embryo Production System (SisEmbrio). Clinics are since required to register in the system and submit reports on the production of human embryos obtained by IVF.

According to ANVISA, 141 services reported 44,705 assisted reproduction cycles and the cryopreservation of 100,380 embryos (70% of them in clinics located in Southeastern Brazil) in 2019, resulting in a 13% increase in the number of frozen embryos compared to 2018 (ANVISA, 2019). IVF clinics use websites and other marketing tools to educate prospective and existing patients and to advertise their treatment offerings. Web-based approaches have the advantage of being easily and readily accessed by individuals worldwide and may present a unique opportunity to educate people and increase knowledge on reproductive health and available treatments (Daniluk & Koert, 2015).

The Society for Assisted Reproductive Technology (SART) and the ASRM have issued guidelines for advertising. These guidelines define how clinics offering Assisted Reproductive Technology (ART) should advertise, market, and report their results. Adherence to these recommendations is an essential requirement for SART membership of United States ART providers (Practice Committee of the Society for Assisted Reproductive Technology and the American Society for Reproductive Medicine, 2004a;b).

In Brazil, however, there are no specific guidelines regarding advertising and marketing for assisted reproduction programs. Therefore, ART clinics must follow the Brazilian Medical Council (Conselho Federal de Medicina, CFM) guidelines for advertising and use of online resources and social media.

Our study evaluated the websites of Brazilian fertility clinics listed in the 11^th^ SisEmbrio Report of 2017 for compliance with the 2004 ASRM and the CFM guidelines for advertising and looked into the general features of their websites and social media presence.

## METHODS

In April 2020, we performed a cross-sectional online evaluation to gather data about the websites of fertility clinics registered with ANVISA listed the 11^th^ SisEmbrio Report (ANVISA, 2018). The report was used to identify clinics and their respective websites. Three of the authors (CKN, MCLM and PFF) collected the data, which was verified by another author (MMC). The data available on the SisEmbrio report included: State in which the clinic was located, number of IVF cycles per State, number of harvested oocytes, and number of transferred and discarded embryos.

The evaluation criteria for each website was based on the 2004 ASRM guidelines for advertising (Practice Committee of the Society for Assisted Reproductive Technology and the American Society for Reproductive Medicine, 2004a;b): 1) the publication of IVF success rates; 2) the presence of additional data to support the published success rate; 3) the presence of advertising/marketing that ranks or compares clinics or practices based on success rate (i.e., comparison marketing); 4) the presence of live birth rates; 5) the method used to calculate live birth rates; 6) live birth data appropriate for the time period being reported; 7) success rates according to age; 8) success rates according to diagnosis; 9) identification of terms comprising the numerator and denominator of the reported success rates; 10) disclosure of investigational or experimental nature of an advertised procedure.

Compliance with CFM guidelines for advertising and use of online resources and social media was also assessed: name of the practice director visible on the website with respective council number; no exhibition of information on costs of treatments; no photos of patients nor success stories with identified patients. In addition, the general characteristics of fertility clinic websites and their social media presence were also assessed. The following data were obtained: State in Brazil where the clinic is located; type of practice (private vs. public); and number of cycles registered. Websites were also surveyed for a variety of additional data including types of treatment offered, information on the team of physicians and associated healthcare professionals, presence on social media, and any other advertised content.

No ethical approval was required as we used only publicly available information and our evaluation did not disclose information that might allow the identification of clinics.

## RESULTS

**Figure 1:**
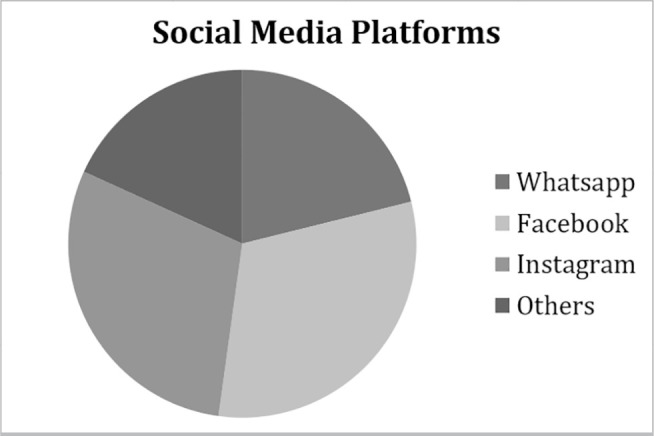
Clinic social media presence.

All 161 SiSEmbrio-registered clinics were evaluated (Table 1); 153 (95.0%) had functional websites, most were private clinics, and only seven were public clinics. Social media presence was as follows: 87 (54.03%) were on WhatsApp; 128 (79.5%) were on Facebook; and 122 (75.8%) were on Instagram. Seventy-five (46.6%) were present in other social media platforms including YouTube, LinkedIn, and Twitter (Figure 1). Regarding CFM recommendations (Table 2), only 49 (30.4%) published the name of a registered director, 85 (52.8%) showed patient photos on their websites or social media accounts along with success stories. None of the clinics published prices, and only one offered an exclusive treatment. Fifty-four published success rates (33.5%) and only 19 (11.8%) used their own success rates, whereas seven (4.3%) showed success rates by age (Table 3). No clinic showed live-birth rates. All clinics offered IVF, and 139 (86.3%) explained the procedure to patients. Social fertility preservation and oncofertility were both offered by 119 (73.9%) clinics. Preimplantation genetic diagnosis was available in 85 clinics (52.8%), oocyte donation in 88 (54.7%), surrogacy in 48 (29.8%), and semen donation in 45 (27.9%); only 36 clinics (22.4%) had an andrologist.

**Table 1:** Summary of the data reported in SisEmbrio regarding number of cycles, production of cells (oocytes) and embryos per Brazilian State in 2017 (ANVISA, 2018)^.^

**State**	**Number of** **clinics**	**Number of** **cycles**	**Number of produced oocytes**	**Number of transferred embryos**	**Number of discarded embryos**
AM	2	113	1,094	241	81
BA	3	1,226	11,245	2,074	2,479
CE	4	810	6337	1,955	229
DF	4	1,048	10,161	2,000	2,371
ES	3	498	4,026	1,060	608
GO	4	962	8,310	2,535	1,289
MA	2	145	1,465	424	137
MG	19	3,700	33,530	8,289	6,162
MS	1	119	1,479	345	496
MT	2	507	5,452	1,201	907
PA	2	334	2,786	355	583
PE	3	940	8,969	1,900	1,901
PI	1	129	1,487	352	252
PR	15	2,305	18,679	4,613	3,081
RJ	10	3,004	27,715	5,380	4,583
RN	3	198	1,836	443	740
RS	8	2,652	25,538	5,017	6,250
SC	7	1,038	8,976	2,077	1,594
SE	1	155	1,483	498	147
SP	51	16,357	160,315	28,159	31,962
TO	1	67	575	73	7
Total	146	36,307	340,458	68,891	65,689

**Table 2:** Data published on clinic websites according to CFM guidelines.

**Data published on clinic websites**	**Number of clinics**
Name and registration number of the clinic’s director	49 (30.4%)
Patient photos and success stories (either on websites and/or social media accounts)	85 (52.8%)
Cost of treatment	0
Exclusive treatment offerings	1 (0.6%)

**Table 3:** Success rates published on clinic websites.

**Success Rates**	**Number of clinics**
Overall success rate	54 (33.5%)
Own success rate	19 (11.8%)
Success rates by age	7 (4.3%)
Live Birth Rate (LBR)	0

The clinics included in Table 1 are the ones that informed their production numbers in terms of number of cycles, production of cells (oocytes) and embryos. Such data allowed the evaluation of Brazil’s average production numbers and supported comparisons between different regions of the country. Since we aimed to provide a qualitative analysis of fertility clinic websites, we searched the tables that contained information about the State in which the clinics were located and their commercial names to further look into their social media presence and websites. Therefore, this study comprised the data from the 161 clinics listed in SisEmbrio that published production data.

## DISCUSSION

In Brazil, most fertility practices are private and have functional websites as well as strong social media presence. Most clinics are in the Southeast region of the country, but as IVF became more popular and the CFM resolutions opened new opportunities for family building, clinics opened all over the country. Unfortunately, only a few follow current CFM guidelines.

There is no denying that the Internet is increasingly seen as a reliable source of information for people needing fertility care. At the moment, there are no specific rules for Brazilian fertility practices regarding advertising, so clinics turn to the CFM guidelines. Regarding CFM recommendations, only a third of the clinics (30.4%) showed the name of a registered director on their websites and a substantial portion (52.8%) pictured patient photos either on their websites and/or social media accounts along with success stories, which is not allowed according to CFM guidelines. Although these are simple and straightforward rules, most clinics failed to comply with them. No clinic announced prices on their websites and only one offered an exclusive treatment.

Published data shows that women believe the Internet is a reliable source for both information and support regarding their infertility (Kahlor & Mackert, 2009; Hammarberg *et al*., 2017). Apparently, infertile women use the Internet to complement rather than to substitute medical advice (Slauson-Blevins *et al*., 2013). Men also use online resources to seek support regarding infertility (Hanna & Gough, 2018). Having an online presence with a good reputation in social media also appears to influence how people choose a fertility clinic (Marcus et al., 2005; Pan *et al*., 2019). Undergoing IVF still presents physical, psychological, and financial challenges. In Brazil, most IVF centers are private and costs are high, therefore online presence is almost mandatory for any fertility practice to advertise their treatments and become known in a highly competitive market.

All clinics offered IVF, which is by far the most popular treatment, and 131 explained the procedure to patients. Most clinics provided information on social fertility preservation (78.8%) and oncofertility-related treatments (75.7%). Information on other ART procedures such as preimplantation genetic testing (52.7%), oocyte donation (41.6%), surrogacy (31.6%) and semen donation (27.3%) were also available, although not universally. Exposure to educational material online may change misconceptions that people have about the timing of parenthood, mainly for women who have a reduced window of opportunity, and may increase fertility and ART awareness (Daniluk & Koert, 2015). As for the so-called “add-ons”, we did not include such analysis in our aims, though we believe that websites should contain current evidence-based information about these procedures. According to the Human Fertilization and Embryology Authority (HFEA), such additional treatments have not been adequately evaluated and should not be routinely offered to patients. The HFEA made a list of 12 items including assisted hatching, artificial egg activation, preimplantation genetic testing for aneuploidies PGT-A), elective freeze-all cycles, embryo glue, and intrauterine culture, to name a few, and categorized them using a traffic-light rating system in an attempt to guide patients. For the record, no add-on was given the green light (HFEA, 2021). Clinic websites should therefore provide scientifically sound information on such procedures, including their possible influence on IVF success rates.

We did not analyze the quality or the scientific accuracy of the information provided in these websites, which is both a limitation of our study and a problem found in other countries regarding procedures such as oocyte cryopreservation. Available online information may be inaccurate and might not adequately reflect current evidence-based recommendations (Avraham *et al*., 2014; Shao *et al*., 2020). Medical doctors and clinics must provide updated and evidence-based guidance and sound information to patients.

Only less than a third of the clinics published success rates and very few (11.8%) reported their own success rates, whereas only seven (4.3%) showed success rate broken down age. This is rather odd, as studies show that one of the main factors that affected the choice of initial and subsequent IVF centers was the success rate of a specific fertility practice and the quality of the service provided. Being recommended by a medical doctor and distance from home are also relevant factors affecting patients’ choice of IVF clinic (Marcus *et al*., 2005).

Social media presence was strong, with Facebook (79.5%) and Instagram (75.8%) emerging as the most popular platforms. WhatsApp, YouTube, LinkedIn, and Twitter were also utilized by almost half of the clinics. Presence in social media is not only useful to provide information to patients, but might also help individuals in need of psychological and social support. This is in fact vital for people undergoing IVF (Sormunen *et al*., 2020) and patients may feel more comfortable contacting their fertility clinics through their social network accounts. Guidelines on social media participation for healthcare providers in Brazil are still shy and focus only on a few aspects. There is an urgent need to evaluate and develop detailed comprehensive guidelines. In the USA, SART member clinics are expected to comply with the guidelines to enhance patient care and minimize the commercial aspect related to IVF as well as maintain collegiality and a respectful relationship among fertility practices (Jain *et al*., 2021).

Online Information provided by fertility clinics in Brazil is heterogeneous. A significant proportion of the websites of SisEmbrio-registered fertility clinics do not follow some aspect of ASRM and CFM guidelines for advertising. As websites and social media are widely used by patients to obtain health information, increased dissemination and awareness of the guidelines is highly recommended. In addition, as couples use success rates to choose a clinic, Brazilian practices should attempt to publish their own pregnancy rates according to age and diagnosis. Further studies should concentrate on the quality of the online content, as well as the impact of social media on fertility care.
